# Refinement of Animal Experiments: Replacing Traumatic Methods of Laboratory Animal Marking with Non-Invasive Alternatives

**DOI:** 10.3390/ani13223452

**Published:** 2023-11-09

**Authors:** Ilya Klabukov, Victoria Shestakova, Olga Krasilnikova, Anna Smirnova, Olga Abramova, Denis Baranovskii, Dmitri Atiakshin, Andrey A. Kostin, Peter Shegay, Andrey D. Kaprin

**Affiliations:** 1 National Medical Research Radiological Centre of the Ministry of Health of the Russian Federation, 249036 Obninsk, Russia; 2 Obninsk Institute for Nuclear Power Engineering, National Research Nuclear University MEPhI, 249039 Obninsk, Russia; 3 Department of Urology and Operative Nephrology, Patrice Lumumba Peoples Friendship University of Russia (RUDN University), 117198 Moscow, Russia; 4 Russian Laboratory Animal Science Association (Rus-LASA), 119992 Moscow, Russia; 5 Scientific and Educational Resource Center for Innovative Technologies of Immunophenotyping, Digital Spatial Profiling and Ultrastructural Analysis, Patrice Lumumba Peoples Friendship University of Russia (RUDN University), 117198 Moscow, Russia

**Keywords:** animal experiment, animal marking, animals, animal welfare, bioethics, ethics, mouse, rat, rodent

## Abstract

**Simple Summary:**

To achieve the highest ethical standards in laboratory research, animal discomfort and the risk of health complications at the identification stage should be minimized. This requires the development of new bioethical yet reliable identification methods or the use of a combination of established non-invasive methods.

**Abstract:**

Reliable methods for identifying rodents play an important role in ensuring the success of preclinical studies. However, animal identification remains a trivial laboratory routine that is not often discussed, despite the fact that more than 6 million rodents are used in animal studies each year. Currently, there are extensive regulations in place to ensure adequate anesthesia and to reduce animal suffering during experiments. At the same time, not enough attention is paid to the comfort of rodents during routine identification procedures, which can be painful and cause some complications. In order to achieve the highest ethical standards in laboratory research, we must minimize animal discomfort during the identification phase. In this article, we discuss traumatic methods of identification and describe several painless methods for marking in long-term experimental studies. The use of non-traumatic and non-invasive methods requires the renewal of marks as they fade and additional handling of the rodents. Laboratory personnel must be trained in stress-minimizing handling techniques to make mark renewal less stressful.

## 1. Introduction

The individual marking of animals and avoidance of misidentification are of great importance for ensuring the success of preclinical studies. Currently, appropriate anesthesia and the reduction in laboratory animal suffering during experiments are widely regulated. However, not enough attention is given to minimizing rodent discomfort and pain during marking, although a wide range of different methods of animal marking is available to researchers and vivarium staff [1,2]. Animal marking remains a trivial laboratory routine that is not often discussed despite the fact that more than 6 million rodents are used in animal studies annually [1]. Despite the usual use of the cage marking approach, individual rodent marking is often required to provide social housing or to carry out experiments involving the simultaneous participation of several animals.

It is well known that all marking techniques are brief procedures restraining the animals, resulting in some degree of discomfort or pain. Since these procedures are performed on a huge number of rodents, even small improvements in the procedures can lead to a significant improvement in the overall effect. Ear punching and notching, toe clipping, and tattooing are frequently used for the permanent marking of laboratory rodents. At the same time, research on the well-being of animals showed that ear punching and tagging may cause pain [3,4]. Following an aseptic technique is a prerequisite for traumatic/invasive methods of marking; however, since these methods are characterized by some degree of physical intervention into the animal body, there may be a risk of infection [5]. Thus, Chen et al. suggested that ear tagging, punching or notching, tattooing, toe clipping, and microchipping may have a potential for infection [6]. Not painful and non-invasive methods, including fur/skin dyeing or marking with a permanent marker, are more relevant from an ethical point of view since they allow for the avoidance of complications and pain related to some traumatic and invasive methods [7]. Non-permanent methods of marking, such as the use of dyes, markers, fur cutting, or shaving, are widely used for animal identification in short-term experiments. These marks are typically applied externally and require periodic renewal to maintain their visibility. At the same time, concerns regarding the reliability of non-invasive marking methods in long-term experiments, as well as concerns regarding additional stress related to the renewal of non-invasive/non-permanent marks, hamper their wider application.

Since rodent marking is an essential part of laboratory research, additional attention should be given to non-invasive marking methods to ensure animal welfare. In this review, we aimed to describe a wide range of marking methods in order to raise awareness of possible ethical non-invasive options. Importantly, we also highlight ways to increase the reliability of non-invasive methods and the possibility of implementing non-invasive marking in long-term experiments with the use of stress-minimizing rodent handling.

## 2. Classification of Methods Used for Rodent Marking

According to the degree of intervention to the bodies of laboratory rodents, we suggest classifying all marking methods into traumatic, invasive, and non-invasive.

### 2.1. Traumatic Methods of Rodent Marking

Traumatic methods are associated with the disruption of animal bodily integrity and include ear tagging, punching, and notching, as well as toe clipping.

Some guidelines recognize that particular traumatic methods of individual identification should be used only when absolutely necessary or when no other forms of marking are possible or feasible [8,9], with the presentation of substantial and convincing justification. When a rodent is marked with the use of traumatic methods, it is critical to consider how this may affect the behavior and well-being of the animal in both the short and long term [10].

An important issue to be mentioned is that ear tagging may create an entry portal for pathogens and result in staphylococcus infection, and metal tags may cause auricular chondritis due to the release of iron and copper ions from the tag in mice [5,11]. Complications from ear punching in laboratory mice were also reported [12]. Ear notching has been associated with increased mean arterial blood pressure in rats, suggesting that it may be painful [13]. Ear punching was also associated with signs of pain and anxiety in mice [14].

Toe clipping is one of the widely used marking methods that additionally allows for the collection of biomaterials for genotyping. Some studies reported neither significant corticosterone (pain and stress marker) elevation in 7-day-old mice after toe clipping [15], nor a higher percentage of vocalizing or urinating newborn mice compared to the control group [16]. However, other sources indicate that toe clipping may be painful and may bear potential for infection if aseptic techniques are not followed [7,8]. The characteristics of various traumatic and invasive methods of rodent marking are presented in Table 1.

### 2.2. Invasive Methods of Rodents Marking

Invasive methods do not disrupt animal bodily integrity but still comprise procedures when the body is injected with an ink or implanted with a special device. Invasive methods include tail, toe, and ear tattooing, and the implantation of transponders for Radio Frequency Identification (RFID). We also include freeze marking in this group of methods since it affects the pigment-producing function of melanocytes in rodent skin.

The tattooing may be performed by using special electronic tattooing machines or manually. Some reports showed that tail tattooing caused significant tail inflammation in mice, as well as agitation and anxiety [4], and the potential for infection was also noted in regard to toe tattooing [6].

Freeze marking relies on the ability of low temperatures to destroy pigment-producing cells in the animals’ skin, resulting in fur discoloration. It provides permanent identification, but can only be used on dark-coated animals and can cause scarring in the case of excessively prolonged cold impact [18].

Despite being one of the most advanced and sophisticated rodent marking systems that additionally allows for the tracking of animal behaviors [20], the implantation of transponders for RFID has a number of disadvantages. Subcutaneous transponder implantation was shown to be the most stressful method of marking compared even to toe clipping and toe tattooing in newborn mice, as suggested by vocalization and urination [16]. It is of the utmost importance to mention that reports on transponder rejection and provocation of tumors in rodents have been published [21].

### 2.3. Non-Invasive Methods as Front-Line Approaches for Rodent Marking

Non-invasive methods are of the highest ethical relevance since they require only painless manipulations, such as fur clipping, fur shaving, fur or skin dyeing, bleach marking, or biometry based on ear blood vessel patterns (Table 2) [14,22,23].

However, there are concerns that non-invasive marking methods are less reliable, which may result in misidentification or require additional handling for repeated marking. We suppose that the use of a combination of several non-invasive marking methods (for example, a combination of fur bleaching with tail marking with permanent marker) may enhance the robustness of non-invasive rodent marking (Table 2). A combination of different types of non-invasive marks can reduce the risk of misidentification when one type of mark fades over time. Applying two different types of non-invasive marks with a certain time interval between them can ensure the presence of either two marks or at least one mark in case of fading.

One of the principal questions is whether non-invasive methods may be modified to allow reliable individual identification in long-term experiments and replace invasive and traumatic methods. Even though non-toxic markers that do not disappear within 6–12 weeks are commercially available [24,25], the renewal of marker signs, dyes, or bleach marks in longer-term studies may be required. Repeated handling and restraint for the renewal of marks may cause stress in rodents; however, following rules of stress-minimizing handling (described below in Section 3) may partially solve this problem. An important consideration is that the renewal of non-invasive marks may help to avoid invasive and traumatic marking methods associated with possible complications and pain (Table 1).

Even ethically acceptable non-invasive methods of individual animal marking may have limitations. The odor of markers used for tail identification may be aversive to rats [26]. In addition, black-marked domestic fowl received more aggression in the form of threats from other birds and had lower body mass [27]. More studies are needed to evaluate the impact of different dyes on social perception in rodents since it may impact the results of experiments. The use of fluorescent and odorless dyes invisible to the animals may represent a possible solution [28]. General considerations with regard to non-invasive marking also include the need to use dyes that do not penetrate the skin in order to avoid an impact on the study results [2].

The use of a system for the biometric identification of rodents based on patterns of ear vessels has also been reported [23]. Biometric identification involves capturing images of unique patterns of ear blood vessels and further identifying individual animals based on these patterns [23]. Further development of the biometric approach may facilitate the recognition of rodents in long-term experiments and help to implement this innovative technique in laboratory practice.

## 3. Stress-Minimizing Handling of Rodents during Marking Procedures

One of the concerns arising in respect to ethical non-invasive marking methods is the potential need for more frequent handling and restraining, which may cause additional stress in animals. Indeed, it was shown that handling results in elevated levels of corticosterone in mice, suggesting increased stress [15]. Even though modern markers and dyes may possibly last on rodent fur for 6–12 weeks [24,25], non-invasive marks should be refreshed in long-term experiments, which emphasizes the need to highlight rules for stress-minimizing rodent handling. The provision of stress- and anxiety-minimizing handling is of great importance since it helps to reduce experimental noise that may interfere with study results, especially in behavioral experiments [29].

The general rule is to handle animals calmly yet firmly, so that they become accustomed to human contact without feeling threatened or stressed. The habituation of mice to the experimenter also has a positive effect on the reduction in stress levels from handling [30]. Training rodents by familiarizing them with different handling techniques is of great importance. Thus, rodents can be familiarized with non-aversive tunnel or cup handling with low time consumption during cage cleaning [31]. It is also important to provide positive reinforcement when training animals by offering rewards such as treats after the successful completion of tasks.

A substantial body of research is devoted to the investigation of the impacts of different handling techniques on anxiety- and stress-like behavior in laboratory rodents. Thus, it was shown that tunnel handling resulted in lower anxiety in comparison to tail handling [4,32]. Some authors also suggest that using tunnels familiar to mice due to the presence in their home cage may be a valuable tool for reducing handling stress [32].

Finally, it is essential that handlers are properly trained in how to handle and restrain mice and rats with minimal stress to the animals. This includes learning how to recognize signs of distress in rodents (e.g., grimacing, vocalization and urination) as well as understanding how different types of restraint devices should be used correctly without causing pain, harm, or discomfort [33]. Training models may be used for the practice of fast and stress-minimizing handling, restraint, and marking to encourage proficiency and reduce stress and discomfort in animals.

## 4. Discussion

Approaches to the minimization of laboratory animal suffering and the implementation of the 3Rs principles are being widely discussed [34,35]. Improving the welfare of animals associated with marking is an important part of the Refinement principle [36], as some traumatic and invasive methods may not only cause pain and discomfort, but also health complications [11]. The development of novel non-invasive but reliable marking methods or readiness to refresh non-permanent marks in long-term studies represent real steps towards the Refinement of animal experiments.

From an ethical point of view, standards of working with laboratory animals should emphasize the importance of non-invasive marking methods. In reality, this task is closely related to the development of new methods of reliable non-invasive marking that could ensure long-term identification. Biometric identification, as described above, represents a promising example of a non-invasive method of permanent identification [23]. Thus, further development and wider application of biometry-based technologies are needed.

Nowadays, commonly used non-invasive marking methods are non-permanent and require the renewal of marks in long-term experiments, which means additional handling and restraint. Although this approach seems more ethical, the comprehensive training of staff in stress-minimizing handling and restraint techniques is required.

It is important to highlight several factors that may influence the longevity of non-invasive, non-permanent marks. These factors may include environmental conditions, animal behaviors, and marking techniques. Environmental factors, such as sunlight exposure and abrasion, can accelerate the fading or removal of marks. Animal behaviors, such as grooming or rubbing against objects, can also affect mark durability. In addition, the choice in marking technique and materials used can have a significant impact on mark longevity.

The length of the study period determines the number of times that marks need to be refreshed; therefore, longer studies may require more frequent refreshing to maintain identification accuracy. The visibility of the mark should be evaluated periodically to determine the need for refreshing. This can be achieved through visual inspections, remote sensing technologies, and cameras. Monitoring the rate of mark deterioration helps to estimate the optimal refresh frequency. The regular assessment of temporary mark legibility and durability allows researchers to adjust the refreshment schedule accordingly.

**Figure 1:**
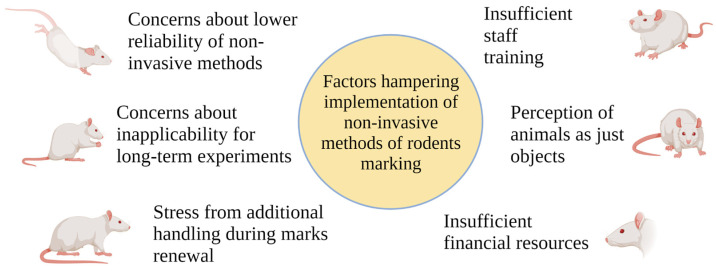
Factors hampering implementation of non-invasive methods of rodent marking. Created with BioRender.com.

We hypothesized possible factors hampering the implementation of non-invasive methods of rodents marking (Figure 1).

1. Concerns about the lower reliability of non-invasive methods is one of the main reasons for the lack of wider use of non-invasive methods. Indeed, the dyes used for marking may fade or may be washed off during grooming. However, this fear can be overcome with constant monitoring of the animals, refreshing the dye as it fades, and the use of a combination of non-invasive methods.
2. Concerns about inapplicability for long-term experiments: Marker and dye marks may fade over time, and shaved or bleached hair can grow back. However, the renewal of non-invasive marks using stress-minimizing handling and restraint techniques may allow non-invasive marking in long-term experiments.
3. Stress related to additional handling during the renewal of non-invasive non-permanent marks: The problem of handling-related stress in rodents is widely recognized. At the same time, non-aversive methods of rodent handling, such as tunnel handling, may reduce stress. There is a need to conduct long-term studies dedicated to the question of welfare in rodents receiving traumatic/invasive permanent marks in comparison with rodents receiving non-invasive marks and more frequent handling.
4. Insufficient financial resources are one of the fundamental causes that limit the implementation of best laboratory practices. The use of ethical non-invasive marking methods requires additional time for the refreshment of dyes or bleach marks, which implies additional working hours for laboratory staff that need to be paid. The development and implementation of novel methods based on biometry also requires financial support.
5. Insufficient staff training: The problem of insufficient staff training is closely related to the lack of financial resources that would allow for the education and familiarization of specialists with ethical and novel approaches to animal marking. Moreover, hiring additional special staff dedicated solely to animal care and support with experimental procedures would reduce the amount of work for research staff and help to achieve higher standards of animal welfare.
6. Perception of animals as just objects: In some cases, laboratory rodents are perceived only as tools to achieve a scientific goal without prioritizing their welfare [37]. In such cases, the implementation of high ethical standards may be lacking. However, rodents are also living beings experiencing pain, suffering, and discomfort. The value of animal life is equal to the value of human life, especially considering that many discoveries in science and medicine were made with the self-sacrificing help of laboratory rodents.

The use of non-invasive methods of rodent marking may be promoted by raising awareness of the feasibility of such methods if they are applied with due precautions or in a combined manner. Bioethical education among researchers may also aid in changing perceptions of laboratory animals and to raise empathy toward them. Legal regulations regarding acceptable methods of animal marking play an important role in the development of a culture of applying a particular method. Regulations governing animal research should contain clear rules regarding choosing particular types of marking with a focus on the most ethical approaches. A list of exemptions from the ‘most ethical marking rule’ should be clearly defined and explicitly establish cases where it is possible to use invasive or traumatic marking methods. Legal regulations regarding animal marking should be known to all laboratory staff, and compliance with such rules should be rigorously monitored.

To summarize the above, in striving to achieve the highest ethical standards, researchers should minimize rodent discomfort, pain, and possible health complications even at the marking stage. However, this task requires a complex approach that includes changing researchers’ perception of animals, familiarity with the possibility of using non-invasive marking methods with due precautions, and the development of new reliable non-invasive marking techniques and associated marking reagents. The most difficult and long-term tasks include achieving higher financial support to provide training for laboratory staff and payments for the additional time required for the renewal of non-invasive marks, as well as the development of novel methods of rodent marking.

The use of non-invasive marking methods may help to avoid health complications related to some methods of traumatic and invasive marking (e.g., infection and tumor formation). Importantly, non-invasive methods allow the avoidance of pain at the marking stage. However, one of the serious limitations of non-invasive marking is represented by the need for additional handling for mark renewal, which bears the potential for additional stress. Novel studies are needed to compare long-term welfare parameters (stress levels and health complications) in rodents marked in a traumatic/invasive manner and in rodents receiving non-invasive marks and stress-minimizing handling required for mark renewal.

## 5. Conclusions

The reduction in rodent discomfort, pain, and health complications even at the marking stage is an important bioethical task that does not require less attention than the reduction in animal pain during experiments. The use of non-invasive painless methods of marking is limited due to fears of the unreliability of the method. In this paper, we described various non-invasive methods of rodent marking and ways of improving their reliability. We aimed to raise awareness of the possibility of painlessly marking rodents without the fear of mixing up individual animals. There is a need to spread knowledge on painless non-invasive methods of marking.

There is a need for the development of new bioethical yet reliable methods of identification. At the moment, we suggest the use of a combination of already established non-invasive methods to avoid concerns about mixing up individual animals. The renewal of non-invasive temporary marks in long-term experiments also seems a more ethical option compared to traumatic and invasive marking; however, rules of stress-minimizing handling and restraint should be followed. Using a combination of non-invasive marking methods and renewal of marks as they fade requires more time from laboratory staff, but such efforts are justified, given that animals make a tremendous contribution to research and deserve the most humane treatment. Additional studies are required to compare welfare in rodents that receive traumatic/invasive marks and in rodents receiving non-invasive marks and additional handling for marks renewal.

## Figures and Tables

**Table 1:** Methods used for animal marking.

№	Method of Animal Marking	Advantages	Disadvantages	References
Traumatic methods				
1	Ear notching/punching	1. Simple and low-cost. 2. Provides long-term identification and allows genotyping.	1. May cause pain. 2. Possible complications. 3. Fights between animals may cause damage to punches/notches and complicate identification.	[2,3,12,13,14]
2	Ear tagging	1. Inexpensive. 2. Wide variety of types and colors of tags allow fast animal identification. 3. Long-term identification.	1. May cause pain, infection, and inflammation. 2. Tag may be caught in a cage, leading to ear damage. 3. Tags may be lost. 4. Requires staff training to insert the tag in the correct spot.	[4,5,11,17]
3	Toe clipping	1. Simple and low-cost. 2. No special equipment needed. 3. Provides permanent identification and allows genotyping.	1. May be painful. 2. Infection may occur if aseptic techniques are not followed.	[7,8]
Invasive methods				
1	Tattooing	1. Long-term identification. 2. May be performed on neonatal pups. 3. Micro-tattoos may be used to reduce stress.	1. Special equipment and staff training are needed. 2. May cause infection, inflammation, and additional anxiety. 3. Tattoo may fade or blur, hampering identification and requiring renewal.	[4,6,13,17]
2	Freeze marking	1. Permanent identification. 2. May be performed on different parts of the rodent’s body. 3. Different marking patterns may enhance visibility.	1. Prolonged cold impact may cause scarring. 2. May be used on dark-coated animals only. 3. Working with cryogenic materials may be dangerous and requires staff training.	[18,19]
3	Implantation of transponders for Radio Frequency Identification	1. Long-term identification. 2. Transponders may be read without additional handling of animals. 3. Novel modifications allow tracking of rodent movement and behavior.	1. Expensive, special equipment is needed for tag implantation and reading. 2. Causes stress during implantation. 3. May cause tumors and inflammation or be rejected from the body.	[16,20,21]

**Table 2:** Non-invasive approaches to rodent marking and possible combinations of various methods.

Methods	Pros	Cons	Recommendations
Fur/skin staining with dyes or markers	1. Painless and easy procedure. 2. May be used on rodents of all ages. 3. Both fur and tail may be stained 4. Large marks and patterns are clearly visible and allow easy identification by staff or special software.	1. Dyes may fade over time or due to grooming. 2. Need to conduct daily monitoring to assess the condition of the mark. 3. Need to renew the mark as it fades, especially in long-term experiments. 4. Predominantly may be used on white-furred rodents.	1. When renewing the mark in long-term experiments, use stress-minimizing handling techniques. 2. Use various colors and patterns when marking an individual animal to increase reliability. 4. Novel marker types allow prolonged marking (up to 6–12 weeks). 5. Use non-toxic markers and dyes that are not aversive to rodents and do not cause health problems. 6. Combination of fur and tail staining allows increased marking reliability.
Fur staining with fluorescent dyes	1. Painless and easy procedure. 2. May be used on rodents of all colors. 3. Fluorescent dyes are not visible to rodents and do not cause aggression.	1. Dyes may fade over time or due to grooming. 2. Need to conduct daily monitoring to assess the condition of the mark. 3. Need to renew the mark as it fades, especially in long-term experiments.	1. When renewing the mark in long-term experiments, use stress-minimizing handling techniques. 2. Use various colors and patterns when marking an individual animal to increase reliability.
Bleach marking	1. Painless procedure. 2. Bleach marks and patterns are clearly visible and allow easy identification by staff or special software. 3. Bleach marks are not removed during grooming.	1. May be used on dark-furred rodents only. 2. Prolonged time may be required to achieve fur bleaching. 3. Bleach marks fade as dark hair regrows. 4. Need to renew the mark as hair regrows in long-term experiments.	1. When renewing the mark in long-term experiments, use stress-minimizing handling techniques. 2. Applying several bleach marks and various patterns allows increased reliability. 3. Remove bleach solutions to avoid skin damage. 4. Combination of bleach marking with tail staining allows increased reliability.
Biometry based on ear blood vessels pattern	1. Painless innovative procedure. 2. Provides permanent identification.	1. Requires special devices and software. 2. Identification errors may occur although the risk is low. 3. Various animals may have undistinguishable biometric patterns. 4. Ear damage due to fights or trauma may complicate identification.	1. Combination with tail or fur staining allows increased reliability in case of ear damage due to fights or trauma.
Fur shaving/clipping	1. Painless procedure. 2. Grooming and manipulation do not remove shaving marks. 3. May be performed on rodents of all colors.	1. Temporary marking due to hair regrowth. 2. Need to conduct daily monitoring to assess the condition of the mark. 3. Need to renew the mark as hair regrows in long-term experiments.	1. Larger shaving area increases reliability. 2. Different locations and patterns of shaving may be used. 3. Combination of fur shaving with fur or tail staining allows increased reliability.

## Data Availability

Data are contained within the article.
